# (2*S*)-1,1-Dichloro-2-(2-chloro­phen­yl)-2-(4-chloro­phen­yl)ethane

**DOI:** 10.1107/S160053680804405X

**Published:** 2009-01-14

**Authors:** Tatiana Cantillana, Lars Eriksson

**Affiliations:** a Department of Environmental Chemistry, Stockholm University, SE-106 91 Stockholm, Sweden; b Department of Physical, Inorganic and Structural Chemistry, Stockholm University, SE-106 91 Stockholm, Sweden

## Abstract

The title compound, C_14_H_10_Cl_4_, is easily crystallized while the other enanti­omorph only forms an oil upon crystallization attempts. The title compound has a considerably higher density, ρ ≃ 1.562 Mg m^−3^ compared to the racemic substance, ρ ≃ 1.514 Mg m^−3^. This is supported by the fact there are two inter­molecular halogen–halogen contacts in the title compound compared with only one the racemic compound. The dihedral angle between the two phenyl rings is 76.83 (5)°

## Related literature

For related literature regarding the structure of the racemic compound, see: Arora & Bates (1976 ▶). For related literature on the toxicological effects, see: Allolio & Fassnacht (2006 ▶), Benecke *et al.* (1991 ▶), Bergenstal *et al.* (1960 ▶); Canti­llana *et al.* (2009 ▶).
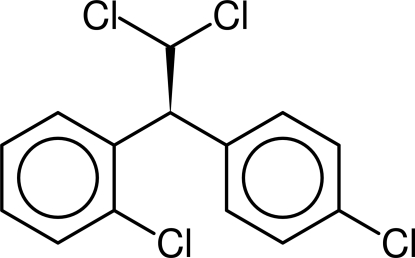


## Experimental

### 

#### Crystal data

C_14_H_10_Cl_4_
*M* *_r_* = 320.02
Monoclinic, 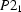

*a* = 6.13530 (10) Å
*b* = 12.0715 (2) Å
*c* = 9.4525 (2) Å
β = 103.5490 (18)°
*V* = 680.59 (2) Å^3^
*Z* = 2
Mo *K*α radiation
μ = 0.85 mm^−1^
*T* = 100 (2) K
0.34 × 0.24 × 0.04 mm

#### Data collection

Oxford Diffraction Xcalibur-3 κ-diffractometer with Sapphire-III CCD
Absorption correction: gaussian (*CrysAlis RED*; Oxford Diffraction, 2008 ▶) *T*_min_ = 0.814, *T*_max_ = 0.968
18569 measured reflections
4258 independent reflections
3935 reflections with *I* > 2σ(*I*)
*R*_int_ = 0.032

#### Refinement

*R*[*F* ^2^ > 2σ(*F* ^2^)] = 0.026
*wR*(*F* ^2^) = 0.060
*S* = 1.01
4258 reflections
164 parameters
1 restraint
H-atom parameters constrained
Δρ_max_ = 0.40 e Å^−3^
Δρ_min_ = −0.31 e Å^−3^
Absolute structure: Flack (1983 ▶), 1755 Friedel pairs
Flack parameter: 0.00 (4)

### 

Data collection: *CrysAlis CCD* (Oxford Diffraction, 2008 ▶); cell refinement: *CrysAlis RED* (Oxford Diffraction, 2008 ▶); data reduction: *CrysAlis RED*; program(s) used to solve structure: *SHELXS97* (Sheldrick, 2008 ▶); program(s) used to refine structure: *SHELXL97* (Sheldrick, 2008 ▶); molecular graphics: *DIAMOND* (Bergerhoff, 1996 ▶); software used to prepare material for publication: *PLATON* (Spek, 2003 ▶) and *SHELXL97*.

## Supplementary Material

Crystal structure: contains datablocks I, global. DOI: 10.1107/S160053680804405X/bq2116sup1.cif

Structure factors: contains datablocks I. DOI: 10.1107/S160053680804405X/bq2116Isup2.hkl

Additional supplementary materials:  crystallographic information; 3D view; checkCIF report

## Figures and Tables

**Table 1:** Selected interatomic distances (Å)

Cl1⋯Cl4^i^	3.4370 (5)
Cl2⋯Cl3^ii^	3.4888 (5)

Symmetry codes: (i) 
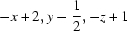
; (ii) 
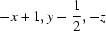
.

**Fig. 1.**
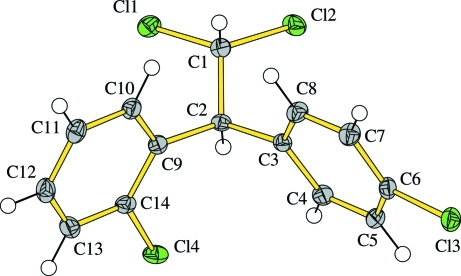
The title compound (I) with displacement ellipsoids at 50% probability with the unique atoms labeled.

## supplementary crystallographic information

### Comment

The title compound is commercially available as a racemate which has been structurally characterized earlier (Arora & Bates, 1976). When purifying and separating the two enantiomers of the racemate, one of the enantiomers, the title compound easily formed crystals while the other enantiomer only formed an oil upon crystallization attempts. A salient feature of the racemic compound o,p'-DDD (Mitotane) is its selective toxicity to the adrenal cortex. It has been used for 40 years for treatment of adrenocortical carcinoma (ACC) (Bergenstal *et al.*, 1960) and Cushing's syndrome (Benecke *et al.*, 1991). The efficacy and potency is however low, and o,p'-DDD treatment is frequently associated with severe side effects (Allolio & Fassnacht, 2006). The differences in toxicity of the two enantiomers of o,p'-DDD and the pharmacokinetics connected with these two compounds has recently been examined in Göttingen mini pigs and will be reported elsewhere (Cantillana *et al.*, 2009).

The crystal structure of (I) shown in Fig. 1 show normal bond distances and angles. The dihedral angle between the two phenyl rings is 76.83 (5)°. Both phenyl rings are planar within 0.01 Å with the Cl3 deviating 0.103 (2) Å from the least square plane calculated from C3→C8 and the Cl4 deviating 0.048 (2) from the least square plane of C9→C14. All four chlorines are involved in the intermolecular Cl···Cl contacts between the different molecules building up a corrugated layer extending in the [010] and [101] directions. The title compound has a considerably higher density, ρ≈ 1.562 g/cm^3^ compared to the racemate, ρ≈ 1.514 g/cm^3^ (Arora & Bates, 1976). A tentative model for the higher density of the pure enantiomer is that it may be a result of the more numerous intermolecular short halogen-halogen contacts.

### Experimental

The title compound was purified from a racemic mixture present in the commercially available product, 1,1-Dichloro-2-(2-chlorophenyl)-2-(4-chlorophenyl)ethane (o,p'-DDD) using high performance liquid chromatography (HPLC), Shimadzu LC-9 A (Kyoto, Japan) equipped with an UV detector, UV100 from Spectra-Physics (Fremont, USA) and a permethylated γ-cyclodextrin column, Nucleodex gamma-PM (250 *x* 10 mm, 5µm, Macherey-Nagel GmbH & Co, Düren, Germany). The detection wavelength was 240 nm and the flow rate was 4 ml/min and injection volume of 200µl. The mobile phase was methanol:water (80:20) and 1% triethylamine:acetic acid (1:2 *v*/*v*). Thin plate-like crystals suitable for X-ray analysis were obtained upon recrystallization from methanol.

### Refinement

The hydrogen atoms were geometrically positioned at C—H distances of 0.95 and 1.00 Å for the aromatic and methine hydrogen's. Both types of hydrogen's were given U(iso) = 1.2*U*_eq_(C). The completeness of the data increases to 0.994 if one cuts the reflection data at 2θ = 50°.

### Crystal data

**Table:**

C_14_H_10_Cl_4_	*F*(000) = 324
*M**_r_* = 320.02	*D*_x_ = 1.562 Mg m^−^^3^
Monoclinic, *P*2_1_	Mo *K*α radiation, λ = 0.71073 Å
Hall symbol: P 2yb	Cell parameters from 11963 reflections
*a* = 6.1353 (1) Å	θ = 3.8–32.1°
*b* = 12.0715 (2) Å	µ = 0.85 mm^−^^1^
*c* = 9.4525 (2) Å	*T* = 100 K
β = 103.5490 (18)°	Plate, colourless
*V* = 680.59 (2) Å^3^	0.34 × 0.24 × 0.04 mm
*Z* = 2	

### Data collection

**Table:**

Oxford Diffraction Xcalibur-3 κ-diffractometer with Sapphire-III CCD	4258 independent reflections
Radiation source: Enhance (Mo) X-ray Source	3935 reflections with *I* > 2σ(*I*)
graphite	*R*_int_ = 0.032
Detector resolution: 16.54 pixels mm^-1^	θ_max_ = 32.2°, θ_min_ = 3.8°
ω scans at different φ	*h* = −9→9
Absorption correction: gaussian (*CrysAlis RED*; Oxford Diffraction, 2008)	*k* = −17→15
*T*_min_ = 0.814, *T*_max_ = 0.968	*l* = −13→14
18569 measured reflections	

### Refinement

**Table:**

Refinement on *F*^2^	Hydrogen site location: inferred from neighbouring sites
Least-squares matrix: full	H-atom parameters constrained
*R*[*F*^2^ > 2σ(*F*^2^)] = 0.026	*w* = 1/[σ^2^(*F*_o_^2^) + (0.0358*P*)^2^] where *P* = (*F*_o_^2^ + 2*F*_c_^2^)/3
*wR*(*F*^2^) = 0.060	(Δ/σ)_max_ = 0.001
*S* = 1.01	Δρ_max_ = 0.40 e Å^−^^3^
4258 reflections	Δρ_min_ = −0.31 e Å^−^^3^
164 parameters	Extinction correction: *SHELXL97* (Sheldrick, 2008), Fc^*^=kFc[1+0.001xFc^2^λ^3^/sin(2θ)]^-1/4^
1 restraint	Extinction coefficient: 0.009 (2)
Primary atom site location: structure-invariant direct methods	Absolute structure: Flack (1983), 1755 Friedel pairs
Secondary atom site location: difference Fourier map	Flack parameter: 0.00 (4)

### Special details

**Table:**

Geometry. All e.s.d.'s (except the e.s.d. in the dihedral angle between two l.s. planes) are estimated using the full covariance matrix. The cell e.s.d.'s are taken into account individually in the estimation of e.s.d.'s in distances, angles and torsion angles; correlations between e.s.d.'s in cell parameters are only used when they are defined by crystal symmetry. An approximate (isotropic) treatment of cell e.s.d.'s is used for estimating e.s.d.'s involving l.s. planes.
Refinement. Refinement of *F*^2^ against ALL reflections. The weighted *R*-factor *wR* and goodness of fit *S* are based on *F*^2^, conventional *R*-factors *R* are based on *F*, with *F* set to zero for negative *F*^2^. The threshold expression of *F*^2^ > σ(*F*^2^) is used only for calculating *R*-factors(gt) *etc*. and is not relevant to the choice of reflections for refinement. *R*-factors based on *F*^2^ are statistically about twice as large as those based on *F*, and *R*- factors based on ALL data will be even larger.

### Fractional atomic coordinates and isotropic or equivalent isotropic displacement parameters (Å^2^)

**Table:**

	*x*	*y*	*z*	*U*_iso_*/*U*_eq_	
C1	0.5723 (2)	0.58327 (13)	0.35281 (15)	0.0153 (3)	
H1	0.4179	0.5513	0.3360	0.018*	
C2	0.5682 (2)	0.70231 (12)	0.40708 (16)	0.0136 (3)	
H2	0.7233	0.7330	0.4219	0.016*	
Cl1	0.75951 (6)	0.50047 (3)	0.48348 (4)	0.02031 (8)	
Cl2	0.66068 (6)	0.58092 (3)	0.18581 (4)	0.02172 (9)	
C3	0.4118 (2)	0.77539 (12)	0.29597 (15)	0.0143 (3)	
C4	0.4896 (3)	0.87540 (13)	0.25332 (16)	0.0178 (3)	
H4	0.6434	0.8943	0.2871	0.021*	
C5	0.3459 (3)	0.94815 (14)	0.16209 (16)	0.0196 (3)	
H5	0.4006	1.0162	0.1335	0.024*	
C6	0.1222 (3)	0.92006 (14)	0.11361 (15)	0.0174 (3)	
C7	0.0412 (3)	0.81928 (14)	0.15077 (17)	0.0197 (3)	
H7	−0.1116	0.7996	0.1141	0.024*	
C8	0.1859 (2)	0.74824 (14)	0.24185 (17)	0.0185 (3)	
H8	0.1310	0.6796	0.2683	0.022*	
Cl3	−0.06289 (6)	1.01373 (3)	0.00690 (4)	0.02230 (9)	
C9	0.5012 (2)	0.70772 (12)	0.55256 (16)	0.0149 (3)	
C10	0.3199 (3)	0.64714 (13)	0.57858 (16)	0.0177 (3)	
H10	0.2424	0.5974	0.5062	0.021*	
C11	0.2503 (3)	0.65801 (14)	0.70765 (17)	0.0192 (3)	
H11	0.1263	0.6162	0.7223	0.023*	
C12	0.3609 (3)	0.72933 (15)	0.81459 (17)	0.0212 (3)	
H12	0.3129	0.7366	0.9026	0.025*	
C13	0.5417 (3)	0.79019 (14)	0.79373 (17)	0.0207 (3)	
H13	0.6192	0.8389	0.8674	0.025*	
C14	0.6088 (2)	0.77922 (13)	0.66334 (16)	0.0157 (3)	
Cl4	0.83309 (6)	0.86142 (3)	0.64058 (4)	0.02012 (8)	

### Atomic displacement parameters (Å^2^)

**Table:**

	*U*^11^	*U*^22^	*U*^33^	*U*^12^	*U*^13^	*U*^23^
C1	0.0144 (6)	0.0158 (7)	0.0172 (6)	0.0000 (5)	0.0071 (5)	−0.0006 (6)
C2	0.0121 (6)	0.0131 (7)	0.0159 (6)	−0.0025 (5)	0.0041 (5)	−0.0019 (5)
Cl1	0.01984 (16)	0.01761 (17)	0.02400 (17)	0.00417 (14)	0.00622 (12)	0.00057 (14)
Cl2	0.02671 (18)	0.02192 (19)	0.02051 (16)	−0.00424 (15)	0.01356 (14)	−0.00507 (14)
C3	0.0158 (6)	0.0140 (7)	0.0139 (6)	−0.0016 (5)	0.0054 (5)	−0.0012 (5)
C4	0.0171 (6)	0.0189 (8)	0.0175 (6)	−0.0054 (6)	0.0042 (5)	−0.0007 (6)
C5	0.0261 (8)	0.0167 (7)	0.0162 (7)	−0.0061 (6)	0.0054 (6)	0.0018 (5)
C6	0.0210 (7)	0.0189 (7)	0.0129 (6)	0.0025 (6)	0.0052 (5)	0.0020 (5)
C7	0.0173 (7)	0.0212 (8)	0.0202 (7)	−0.0024 (6)	0.0039 (6)	0.0019 (6)
C8	0.0171 (7)	0.0174 (7)	0.0206 (7)	−0.0043 (6)	0.0037 (5)	0.0021 (6)
Cl3	0.02784 (18)	0.02156 (19)	0.01717 (15)	0.00582 (15)	0.00459 (13)	0.00309 (14)
C9	0.0156 (6)	0.0138 (7)	0.0154 (6)	0.0041 (5)	0.0034 (5)	0.0005 (5)
C10	0.0205 (7)	0.0166 (7)	0.0172 (6)	0.0015 (6)	0.0070 (5)	0.0021 (6)
C11	0.0186 (7)	0.0203 (8)	0.0205 (7)	0.0034 (6)	0.0081 (6)	0.0048 (6)
C12	0.0263 (8)	0.0224 (8)	0.0163 (7)	0.0089 (7)	0.0077 (6)	0.0029 (6)
C13	0.0258 (8)	0.0189 (8)	0.0158 (7)	0.0070 (6)	0.0020 (6)	−0.0012 (6)
C14	0.0146 (6)	0.0126 (7)	0.0185 (6)	0.0029 (5)	0.0013 (5)	−0.0002 (5)
Cl4	0.01702 (16)	0.01679 (17)	0.02510 (18)	−0.00235 (13)	0.00201 (13)	−0.00440 (14)

### Geometric parameters (Å, °)

**Table:**

C1—C2	1.528 (2)	C7—C8	1.381 (2)
C1—Cl1	1.7831 (15)	C7—H7	0.9500
C1—Cl2	1.7855 (14)	C8—H8	0.9500
C1—H1	1.0000	C9—C14	1.398 (2)
C2—C9	1.526 (2)	C9—C10	1.400 (2)
C2—C3	1.526 (2)	C10—C11	1.390 (2)
C2—H2	1.0000	C10—H10	0.9500
C3—C4	1.392 (2)	C11—C12	1.379 (2)
C3—C8	1.399 (2)	C11—H11	0.9500
C4—C5	1.391 (2)	C12—C13	1.382 (2)
C4—H4	0.9500	C12—H12	0.9500
C5—C6	1.383 (2)	C13—C14	1.394 (2)
C5—H5	0.9500	C13—H13	0.9500
C6—C7	1.390 (2)	C14—Cl4	1.7503 (16)
C6—Cl3	1.7462 (16)		
			
Cl1···Cl4^i^	3.4370 (5)	Cl2···Cl3^ii^	3.4888 (5)
			
C2—C1—Cl1	110.68 (10)	C8—C7—C6	119.01 (14)
C2—C1—Cl2	110.12 (10)	C8—C7—H7	120.5
Cl1—C1—Cl2	108.88 (8)	C6—C7—H7	120.5
C2—C1—H1	109.0	C7—C8—C3	121.31 (14)
Cl1—C1—H1	109.0	C7—C8—H8	119.3
Cl2—C1—H1	109.0	C3—C8—H8	119.3
C9—C2—C3	109.67 (11)	C14—C9—C10	116.49 (13)
C9—C2—C1	111.82 (12)	C14—C9—C2	121.46 (13)
C3—C2—C1	111.69 (12)	C10—C9—C2	121.92 (13)
C9—C2—H2	107.8	C11—C10—C9	121.65 (15)
C3—C2—H2	107.8	C11—C10—H10	119.2
C1—C2—H2	107.8	C9—C10—H10	119.2
C4—C3—C8	118.35 (14)	C12—C11—C10	120.19 (15)
C4—C3—C2	119.83 (13)	C12—C11—H11	119.9
C8—C3—C2	121.69 (13)	C10—C11—H11	119.9
C5—C4—C3	121.07 (14)	C11—C12—C13	120.04 (14)
C5—C4—H4	119.5	C11—C12—H12	120.0
C3—C4—H4	119.5	C13—C12—H12	120.0
C6—C5—C4	119.09 (15)	C12—C13—C14	119.25 (15)
C6—C5—H5	120.5	C12—C13—H13	120.4
C4—C5—H5	120.5	C14—C13—H13	120.4
C5—C6—C7	121.11 (15)	C13—C14—C9	122.37 (15)
C5—C6—Cl3	119.54 (13)	C13—C14—Cl4	117.29 (12)
C7—C6—Cl3	119.33 (12)	C9—C14—Cl4	120.33 (12)
			
Cl1—C1—C2—C9	−57.58 (13)	C2—C3—C8—C7	174.53 (14)
Cl2—C1—C2—C9	−178.02 (9)	C3—C2—C9—C14	−96.67 (15)
Cl1—C1—C2—C3	179.08 (9)	C1—C2—C9—C14	138.85 (14)
Cl2—C1—C2—C3	58.64 (13)	C3—C2—C9—C10	78.99 (17)
C9—C2—C3—C4	107.05 (15)	C1—C2—C9—C10	−45.48 (18)
C1—C2—C3—C4	−128.40 (14)	C14—C9—C10—C11	0.2 (2)
C9—C2—C3—C8	−68.77 (17)	C2—C9—C10—C11	−175.63 (14)
C1—C2—C3—C8	55.78 (18)	C9—C10—C11—C12	−0.3 (2)
C8—C3—C4—C5	1.6 (2)	C10—C11—C12—C13	−0.1 (2)
C2—C3—C4—C5	−174.36 (14)	C11—C12—C13—C14	0.6 (2)
C3—C4—C5—C6	0.1 (2)	C12—C13—C14—C9	−0.7 (2)
C4—C5—C6—C7	−2.1 (2)	C12—C13—C14—Cl4	178.08 (12)
C4—C5—C6—Cl3	176.66 (12)	C10—C9—C14—C13	0.3 (2)
C5—C6—C7—C8	2.3 (2)	C2—C9—C14—C13	176.15 (14)
Cl3—C6—C7—C8	−176.42 (12)	C10—C9—C14—Cl4	−178.44 (11)
C6—C7—C8—C3	−0.6 (2)	C2—C9—C14—Cl4	−2.55 (19)
C4—C3—C8—C7	−1.3 (2)		

Symmetry codes: (i) −*x*+2, *y*−1/2, −*z*+1; (ii) −*x*+1, *y*−1/2, −*z*.
